# Psychometric properties of the Chinese sport emotion questionnaire (SEQ-C) among table tennis athletes in Shandong, China

**DOI:** 10.3389/fpsyg.2026.1795960

**Published:** 2026-03-27

**Authors:** Linghong Liu, Garry Kuan, Yee Cheng Kueh

**Affiliations:** 1 Institute of Physical Education, Jiangsu Normal University, Xuzhou, Jiangsu Province, China; 2 Exercise and Sports Science Programme, School of Health Sciences, Universiti Sains Malaysia, Kota Bharu, Kelantan, Malaysia; 3 Biostatistics and Research Methodology Unit, School of Medical Sciences, Universiti Sains Malaysia, Kota Bharu, Kelantan, Malaysia

**Keywords:** table tennis, adolescent athletes, exploratory factor analysis, confirmatory factor analysis, sport emotion, psychometric properties

## Abstract

**Background:**

The Sport Emotion Questionnaire (SEQ) is a reliable and valid multidimensional instrument for assessing athletes’ pre-competition emotional states. This study aimed to test the Chinese-translated version (SEQ-C) by examining pre-training emotional states of table tennis athletes, using validity and reliability analyses.

**Methods:**

This study employed a cross-sectional survey design using a cluster-based sampling strategy. A total of 670 athletes aged 15–18 years from the Jining Youth Table Tennis Training Base in Jining City, Shandong Province, China, participated in the study; 656 valid responses were retained after data screening. The original English version of the SEQ was translated into Chinese and validated through expert review and pilot testing. Data were collected in September 2024 using a self-administered questionnaire administered via a mixed-mode approach (online and offline), yielding a response rate of 97.9%. Data analysis included a common method bias test, exploratory factor analysis (EFA), and confirmatory factor analysis (CFA). Model fit was evaluated using the standardized root mean square residual (SRMR), root mean square error of approximation (RMSEA), Tucker–Lewis index (TLI), and comparative fit index (CFI). Construct validity was assessed using composite reliability (CR), average variance extracted (AVE), and inter-factor correlations. The study adhered to ethical research standards and was approved by the Human Research Ethics Committee of Universiti Sains Malaysia.

**Results:**

All observed items demonstrated strong factor loadings, confirming that the assumed model, consisting of 22 items grouped into five latent variables, was consistent with the original model. The CFA result indicated a good model fit, as reflected by fit indices: RMSEA = 0.042 (90% CI: 0.037, 0.048), SRMR = 0.026, CFI = 0.971, TLI = 0.966.

**Conclusion:**

The final measurement model retained all 22 items, each deemed acceptable for the target sample. The findings suggest that the SEQ-C is a valid and reliable instrument for assessing the pre-training emotional states of table tennis athletes in Shandong Province, China. Researchers, sport psychologists, and coaches can adopt the SEQ-C framework to effectively capture the unique emotional experiences of Chinese-speaking athletes, thereby providing valuable insights for emotional regulation strategies and psychological interventions within sports settings.

## Introduction

Although emotions are widely recognised as a crucial factor influencing individual behaviour and are closely associated with complex psychological processes such as cognition, motivation, and attitudes (Bandura, 1977; Scherer, 2009), their underlying mechanisms remain a topic of ongoing research. Fredrickson (2001) proposed that emotion is essentially a psychological response triggered by an individual’s cognitive appraisal of a specific event. This response may occur either consciously or unconsciously and can initiate a series of reaction tendencies that manifest across loosely connected component systems, such as subjective experience, facial expression, cognitive processing, and physiological changes” (p. 218). In sport settings, athletes frequently experience a variety of emotions (Lazarus, 2000; Hanin, 2012). These emotions may be positive or negative and are often intense during competition. Such emotional experiences not only influence athletes’ behaviors and performance but may also affect coaches, teammates, and spectators due to the inherently social nature of emotional expression.

In table tennis competitions, athletes continuously win or lose points, which directly affects the likelihood of winning the match. Moreover, athletes often experience considerable psychological pressure during competition. Common challenges include lack of self-confidence, excessive anxiety, and inadequate tactical responses to opponents (Chen et al., 2010). These factors may trigger both positive and negative emotional responses (Sève et al., 2007; Martinent and Ferrand, 2009), making emotional regulation more difficult and potentially impairing athletic performance. Adolescence is widely recognised as a critical developmental stage characterised by heightened emotional sensitivity. This period is crucial for neuronal plasticity (Chen et al., 2010), identity formation (Nelson et al., 2018), and the establishment of behavioural tendencies. As a result, adolescents are more vulnerable to emotional maladjustment. Emotions, therefore, play a central role in youth sport participation (Lewis et al., 2017). In addition to the psychological demands of competition, adolescent table tennis players often face additional stressors such as academic pressure. Previous research has emphasized that effective regulation and management of sport-related emotions are essential for both the psychological well-being and athletic development of adolescent table tennis players (Guo et al., 2025).

Emotions are a fundamental component of athletic performance and have received considerable attention in sport psychology research (Hanin, 2012). From a psychological perspective, sport-related emotions are closely linked to athletes’ cognitive appraisal of situational factors. Specifically, perceived control, competitive pressure, and expectations regarding performance outcomes are key determinants of the type and intensity of emotional states (Jones et al., 2005; Lewis et al., 2017). Over the years, several instruments have been developed to assess emotions in sport and exercise contexts, including the Discrete Emotions Questionnaire (Harmon-Jones et al., 2016), Exercise Induced Feeling Inventory (EFI) (Gauvin and Rejeski, 1993), Subjective Exercise Experience Scale (SEES) (MeAuley and Courneya, 1994), Brunel Mood Scale (BRUMS), Profile of Mood States (POMS) (McNair et al., 1971), and Positive and Negative Affect Schedule (PANAS) (Watson et al., 1988). However, these instruments present certain limitations. Some do not clearly distinguish between emotion and mood states, while others assess only limited emotional dimensions, making them less suitable for capturing the complex and dynamic emotional experiences that occur during competition. To address these limitations, Jones et al. (2005) developed the Sport Emotion Questionnaire (SEQ), grounded in established theories of emotion (Lane and Terry, 2000; Fredrickson, 2001; Jones et al., 2005). The SEQ is a multidimensional tool designed to assess athletes’ pre-competition emotional states. It comprises 22 items across five subscales: Anxiety, Dejection, Excitement, Anger, and Happiness. Because it is specifically designed for competitive sport environments, the SEQ has been widely used in sport psychology research and has demonstrated strong reliability and validity (Vast et al., 2010; Arnold and Fletcher, 2015).

Given the strong psychometric validity and broad applicability of the SEQ, numerous studies have examined its validity across different athletic populations and cultural contexts. The SEQ has been validated in multiple countries and regions, including the United Kingdom (Arnold and Fletcher, 2015), Spain (González-García et al., 2020), Turkey, Germany (Wetzel et al., 2020), Persia (Dehkordi et al., 2020), Portugal (Gomes et al., 2021), and several African countries (Hagan et al., 2022). However, findings from these validation studies have not been entirely consistent. Some researchers have reported discrepancies in the SEQ’s structural validity, reliability, and generalizability (Arnold and Fletcher, 2015; Bayköse and Şakar, 2018; Wetzel et al., 2020). Despite its growing international use, validation studies conducted within Asian contexts remain limited. Existing research has primarily focused on Persian populations in Southwest Asia (Dehkordi et al., 2020), which differ substantially from China and other East Asian countries in terms of cultural values, social norms, and behavioural practices. These cultural differences may influence how athletes experience and express emotions during competition (Hagan, 2021). Therefore, examining the psychometric properties of the SEQ within the Chinese context is both theoretically important and practically meaningful.

Previous studies have reported variations in the psychometric performance of the SEQ across different cultural contexts (Bayköse and Şakar, 2018; Dehkordi et al., 2020; González-García et al., 2020; Gomes et al., 2021; Hagan et al., 2022). These findings suggest that further validation is necessary to ensure the robustness and cross-cultural applicability of the instrument. Additionally, the original developers of the SEQ (Jones et al., 2005) emphasized the importance of continuous validation across various samples. From a cultural perspective, traditional Chinese values may influence how emotions are experienced and expressed. Confucian beliefs emphasizing restraint in emotional expression, Taoist advocacy of inner harmony, dialectical thinking, and collectivist coping strategies may shape emotional regulation processes among Chinese individuals (Hu and Gan, 2008; Wang, 2023). Within this cultural context, adolescent athletes may be more likely to suppress or internalize emotional experiences, adolescent athletes are more likely to suppress or internalize emotional experiences (Yang et al., 2024). Consequently, validation of emotion measurement instruments within specific cultural contexts is of considerable importance. Table tennis is a traditional sport in China, often involves systematic training beginning at a very young age. By adolescence, many athletes reach a stage of competitive maturity, resulting in a highly competitive training environment. These characteristics highlight the importance of reliable psychological assessment tools for youth athletes. The SEQ, as a self-report instrument, is easy to administer and cost-effective, making it suitable for large-scale use in training and pre-competition settings.

In summary, validating the psychometric properties of the SEQ among Chinese adolescent table tennis players helps address the current limitations related to cultural adaptability. It also provides a reliable tool for assessing athletes’ emotional states and subjective experiences in competitive contexts. The findings of this study may offer valuable insights for coaches and trainers in implementing effective emotion regulation strategies and psychological interventions, highlighting the practical importance of the present study.

## Materials and methods

### Participants

This study adopted a cross-sectional survey design to investigate the research variables among youth table tennis athletes. A cluster-based sampling strategy was employed to recruit participants from the Jining Youth Table Tennis Training Base in Jining City, Shandong Province, China. Specifically, athletes were organized into training groups according to their daily training schedules, and all athletes within the selected training groups were invited to participate in the study. Information about the study was disseminated through a promotional poster displayed on the training centre’s notice board. Athletes who expressed interest were provided with a detailed explanation of the research purpose and procedures and were asked to sign an informed consent form before participation.

The questionnaire was distributed and completed approximately 60 min before the start of training. A total of 670 athletes completed the survey. After screening the responses according to the inclusion criteria, 656 valid questionnaires were retained for the final analysis.

The inclusion criteria for participants were as follows: Chinese table tennis athletes (male or female) aged between 15 and 18 years who voluntarily consented to participate in the study. Athletes were categorized into four sport levels: master class, national level 1, national level 2, and without level (more than 5 years of training experience and/or less than 5 years) based on the grading standards of the General Administration of Sport of China, specifically requiring participation in “designated competitions” and achieving “corresponding rankings or results.” According to this system, National Master Class Athletes must achieve outstanding results in international competitions or national top-tier events; National Level 1 Athletes must excel in national competitions or provincial highest-level events; National Level 2 Athletes must attain specified rankings in official provincial-level competitions. Additionally, participants were required to have no family history of serious illness. Of the final sample, 365 participants (55.6%) were male, and 291 participants (44.4%) were female.

When conducting the confirmatory factor analysis (CFA), a large sample size would generally produce more robust solutions and is likely to be replicable (Kyriazos, 2018). According to Hair et al. (2019) a sample size of approximately 500 participants is recommended when analysing models with a large number of constructs. Therefore, the sample size of 656 participants in the present study was considered sufficiently large to support the validity and reliability of the CFA results.

### Questionnaire translation

The SEQ was translated into Chinese using a standard translation–back translation procedure. First, the second and third authors, both professors in sport psychology with extensive experience in questionnaire translation and fluent in both Chinese and English, translated the original English version into Chinese. Next, two independent bilingual translators back-translated the preliminary Chinese version into English (Edunov et al., 2018). The back-translated version was compared with the original English version to ensure semantic equivalence and identify any discrepancies.

Subsequently, a panel of experts in sport psychology, health psychology, and sports training reviewed the translated versions to ensure conceptual consistency and cultural appropriateness of the items. Finally, a pilot test of the finalized SEQ-C was conducted with 20 athletes from the Jining Youth Table Tennis Training Base in Shandong Province, China, to confirm the clarity and applicability of the questionnaire for the target population.

### Data collection

Data collection was conducted in September 2024 at the Jining Youth Table Tennis Training Base, located in Jining City, Shandong Province, China. This study employed a cross-sectional research design, and questionnaire data were collected through both online and offline data collection approaches. The online version of the questionnaire was administered via “Wenjuanxing,” a platform functionally equivalent to Amazon Mechanical Turk. For younger athletes who did not have access to mobile phones, paper-based self-administered questionnaires were distributed to ensure full participation from the target population.

Before questionnaire distribution, the researchers explained this study to participants and sent them an informed consent form with detailed information. Participants would be screened for eligibility by researchers before joining the study. Eligible players were invited to enrol in this study and were required to fill out the informed consent forms and questionnaire. For participants under 18 years of age, assent was obtained from the minors after they were fully informed about the study. In addition, written informed consent was obtained from their parents or legal guardians. To obtain parental consent, the research team first contacted the training team administrators and coaches at the Jining Youth Table Tennis Training Base, who assisted in distributing the study information and consent forms to the athletes’ families. Only participants who provided the appropriate parental or guardian consent, along with their assent, were included in the study. All participants voluntarily completed the questionnaires and returned them on the spot. Researchers reviewed the responses for errors and omissions to minimize the number of invalid questionnaires. Throughout the process, participants could consult researchers for clarification or assistance. A total of 670 individuals completed the questionnaire; however, 14 responses were excluded due to improper completion. Consequently, 656 valid responses were retained, resulting in a response rate of 97.9%. All participants’ information was strictly for the use of this study only. It is strictly prohibited to disclose information to non-participants, and all collected data will be handed over to a dedicated person in the research team for safekeeping after collection.

## Measures

### Demographic

This study included basic demographic information such as age, gender, and skill level. Athletes were classified into master class, national level 1, national level 2, and without level based on the grading standards set by the General Administration of Sport of China.

### Sport emotion questionnaire (SEQ)

The Sport Emotion Questionnaire (SEQ) (Jones et al., 2005) was employed to assess athletes’ emotional states pre-competition, with a greater focus on the positive emotions within the sporting environment (Skinner and Brewer, 2004). It comprises 22 items categorized into five factors: anger, anxiety, dejection, excitement, and happiness. Participants rated each item on a 5-point Likert scale ranging from 0 (not at all) to 4 (extremely), with high scores reflecting positive sports emotions and low scores representing negative sports emotions.

### Ethics approval

The study was conducted in accordance with the Declaration of Helsinki (World Medical Association, 2013) and was approved by the Human Research Ethics Committee of Universiti Sains Malaysia (USM/JEPeM/KK/24030231). Before participation, all individuals were provided with detailed information about the study, and all participants confirmed their understanding of the study information before providing written informed consent. After signing the consent form, participants voluntarily completed the SEQ-C questionnaire and returned it to the researchers. Participants were assured of the confidentiality of their responses and were informed of their right to withdraw from the study at any stage without penalty.

### Statistical analysis

All data were analysed using SPSS 31.0. To assess the potential influence of common method bias (CMB), Harman’s single-factor test was conducted. All measurement items were entered into an exploratory factor analysis using an unrotated principal component solution.

Before conducting confirmatory factor analysis (CFA), exploratory factor analysis (EFA) was performed to examine the underlying factor structure of the translated SEQ-C. The Kaiser–Meyer–Olkin (KMO) test and Bartlett’s test of sphericity were used to assess the suitability of the data for factor analysis. A KMO value greater than 0.7 and a significant Bartlett’s test (*p* < 0.05) indicate that the data are appropriate for factor analysis (Watkins, 2018). The number of factors to retain was determined using the eigenvalue greater than 1.0 criterion, together with examination of the scree plot (Gaskin and Happell, 2014; Watkins, 2018). Principal component analysis with varimax rotation was applied to extract the factors. To ensure adequate convergent validity of the factor structure for the present sample size, factor loadings of at least 0.55 were considered acceptable (Hair et al., 2019).

The measurement CFA model of the SEQ-C was tested using Mplus 8.3. If missing values were present in the dataset, a missing data model was applied. In the absence of missing values, the complete dataset was used for subsequent analyses. For CFA, when the data adhere to the assumption of normality (verified through Mardia’s multivariate skewness and kurtosis tests with *p*-values > 0.05), the maximum likelihood (ML) estimator was employed for model fitting. In such cases, unbiased standardized estimates of path coefficients were reported. If the normality assumption was violated, the robust maximum likelihood (MLR) estimator was used instead. This enhances the robustness of the model against deviations from normality in the data distribution, providing relatively stable parameter estimates.

The hypothesized measurement model included five latent variables (corresponding to the SEQ-C subscales) and 22 observed variables (the SEQ-C items). Factor loadings of 0.40 and above, with a significant p-value, were used as a guide to retain or remove the items from the measurement model (Ford et al., 1986; Hair et al., 2019).

This study conducted a comprehensive evaluation of the model’s validation using multiple fit indices, each required to meet established threshold criteria to determine the adequacy of model fit. Specifically, a good model fit is indicated when the comparative fit index (CFI) and Tucker and Lewis index (TLI) both exceed 0.95, the root mean square error of approximation (RMSEA) is lower than 0.08, and the standardized root mean square residual (SRMR) is lower than 0.08. When these indices simultaneously satisfy the recommended thresholds, the model is considered to demonstrate an acceptable and robust fit to the data (Hair et al., 2019; Kline, 2023).

After establishing the best-fit measurement model, composite reliability (CR) and average variance extracted (AVE) were calculated for each of the five latent factors.

CR was calculated based on Raykov’s method to measure the reliability of the CFA measurement scale (Raykov and Marcoulides, 2016). CR is recommended over Cronbach’s alpha for reliability assessment in CFA models (Raykov, 2001). A CR value of 0.60 or higher is generally acceptable, while an AVE value of 0.50 or higher indicates adequate convergent validity (Fornell and Larcker, 1981; Tseng et al., 2006). Discriminant validity is used to analyse the degree to which one factor is distinct from the other factors (Kline, 2011). Discriminant validity is generally considered adequate when the correlations between factors are ≤ 0.85 (Fornell and Larcker, 1981; Kuan et al., 2019).

## Results

### Common method bias test

The results indicated that four factors with eigenvalues greater than 1 were extracted. The first factor accounted for 43.542% of the total variance, which was below the recommended threshold of 50% (Podsakoff et al., 2003; Fuller et al., 2016), indicating that common method bias was not a serious concern in this study (see Table 1).

**Table 1:** The fit indices used in CFA were presented.

Model fit indices	Cut-off value	Suggested by	Comments
Root mean square error of approximation (RMSEA)	<0.05 (good fit) <0.08 (reasonably fit) <0.10 (poor fit)	Hair et al. (2019); Kline (2023)	
Standardixed root mean square residual (SRMR)	<0.08 (acceptable fit)		
Comparative fit index (CFI)	>0.95 (goode fit), between 0.90 and 0.95 (reasonable fit)	Hair et al. (2019)	For sample size more than 250; (a) If m < 12, CFI/TLI ≥ 0.095 (b) If12 < m < 30, CFI/TLI > 0.92
Tucker-lewis index (TLI)	>0.95 (good fit)		

### Exploratory factor analysis (EFA)

Before conducting CFA, an exploratory factor analysis (EFA) was performed to examine the underlying factor structure of the SEQ-C. The KMO measure of sampling adequacy was 0.937, indicating excellent sampling adequacy. Bartlett’s test of sphericity was statistically significant (*χ*^2^ = 8221.104, *df* = 231, *p* < 0.001), demonstrating that the data were suitable for factor analysis (See Table 2).

**Table 2:** KMO and Bartlett’s test.

KMO measure of sampling adequacy	Bartlett’s test approx. chi-square	*df*	Sig.
0.937	8221.104	231	<0.001

Using principal component analysis with varimax rotation, five factors with eigenvalues greater than 1 were extracted. These five factors collectively explained 68.945% of the total variance, indicating a satisfactory level of explained variance. The scree plot also supported the retention of five factors (See Table 3).

**Table 3:** Total variance explained by extracted factors.

Factor	Eigenvalue	Variance (%)	Cumulative (%)
1	9.579	43.542	43.542
2	1.730	7.862	51.404
3	1.472	6.689	58.093
4	1.252	5.692	63.785
5	1.135	5.160	68.945

The rotated factor loading matrix showed that all items had factor loadings above 0.55 on their respective factors, suggesting a clear and stable factor structure. The distribution of items across the five factors was consistent with the theoretical dimensions of the original SEQ, including anger, anxiety, dejection, excitement, and happiness (See Table 4).

**Table 4:** Exploratory factor analysis of the SEQ-C (rotated factor loadings).

Item	Factor 1	Factor 2	Factor 3	Factor 4	Factor 5
SEQ4			0.795		
SEQ9			0.771		
SEQ14			0.722		
SEQ19			0.718		
SEQ11		0.724			
SEQ21		0.687			
SEQ6		0.728			
SEQ16		0.714			
SEQ1		0.811			
SEQ2	0.782				
SEQ7	0.806				
SEQ12	0.795				
SEQ17	0.721				
SEQ22	0.732				
SEQ3				0.752	
SEQ8				0.755	
SEQ13				0.74	
SEQ18				0.701	
SEQ5					0.738
SEQ10					0.694
SEQ15					0.707
SEQ20					0.717

Factor loadings below 0.40 are suppressed.

### Measurement model SEQ-C

Table 5 shows the demographic characteristics of 656 participants. Among them, 365 (55.6%) were male, and 291 (44.4%) were female. The participants’ ages ranged from 15 to 18 years, with a mean age of 17.1 years (SD = 0.71). Regarding athletic sport levels, 32 participants (4.9%) were master class, 136 (20.7%) as national level 1, and 234 (35.7%) as national level 2. Additionally, 135 participants (20.6%) had more than 5 years of training experience but without sport level, while 119 participants (18.1%) had less than 5 years of training experience.

**Table 5:** Demographic characteristics of participants (*n* = 656).

Characteristic	Category	*n* (%)	Mean (SD)
Gender	Male	365 (55.6)	
	Female	291 (44.4)	
Age (years)	Oveall	—	17.1 (0.71)
	15–16	247 (37.6)	—
	17–18	409 (62.4)	—
Sport levels	Master class	32 (4.9)	—
	National level 1	136 (20.7)	—
	National level 2	234 (35.7)	—
	>5 years of training experience (without level)	135 (20.6)	—
	<5 years of training experience (without level)	119 (18.1)	—

SD, standard deviation.

The SEQ was translated into Chinese (SEQ-C) in strict accordance with the translation procedures, without altering the original intent of the questionnaire. As there were no missing values in the collected responses, the entire dataset was included in the subsequent analysis. Furthermore, the data met the assumption of multivariate normality, as confirmed by Mardia’s multivariate skewness and kurtosis tests (*p*-values > 0.05). Accordingly, the MLR was employed for the CFA.

The outcomes of all the examined models are presented in Tables 6, 7. The hypothesized measurement model of the SEQ-C consists of five latent factors and a total of 22 observed items, with a varying number of items assigned to each factor. The standardized factor loadings for all items are reported in Table 7.

**Table 6:** Goodness of fit indices for SEQ-C.

Model	*χ* ^2^	*df*	*χ*^2^/*df*	RMSEA (90% CI)	SRMR	CFI	TLI
SEQ-C	437.954**	199	2.201	0.042 (0.037, 0.048)	0.026	0.971	0.966

*χ*^2^, Chi-Square; *df*, degrees of freedom; RMSEA, root mean square error of approximation; SRMR, standardized root mean square residual; CFI, comparative fit index; TLI, Tucker–Lewis index.

**Table 7:** Standardized factor loading of the SEQ-C based on CFA.

Factor/item	Factor loadings	Composite reliability	AVE
Factor 1: anger			
Irritated	0.832	0.874	0.634
Furious	0.855		
Annoyed	0.744		
Angry	0.749		
Factor 2: anxiety			
Nervous	0.785	0.873	0.579
Anxious	0.745		
Tense	0.742		
Apprehensive	0.751		
Uneasy	0.780		
Factor 3: dejection			
Upset	0.776	0.898	0.638
Sad	0.875		
Unhappy	0.844		
Disappointed	0.743		
Dejected	0.748		
Factor 4: excitement			
Exhilarated	0.737	0.844	0.575
Excited	0.762		
Enthusiastic	0.765		
Energetic	0.768		
Factor 5: happiness			
Pleased	0.740	0.827	0.544
Joyful	0.707		
Cheerful	0.749		
Happy	0.753		

The results of the analysis showed a good fit to the five-factor model based on the fit indices (RMSEA = 0.042 (90% CI: 0.037, 0.048), SRMR = 0.026, CFI = 0.971, TLI = 0.966). The factor loadings ranged from 0.707 to 0.875 (see Table 7).

### Convergent and discriminant validity

Table 7 illustrates the results of the construct reliability analysis for the hypothesized model. The calculated CR values ranged from 0.827 to 0.898, indicating high reliability of the structure. Also, the AVE values for each factor ranged from 0.544 to 0.638, demonstrating acceptable convergent validity. These findings suggest that the five factors of the SEQ scale exhibit satisfactory construct validity and reliability (Fornell and Larcker, 1981). Furthermore, all inter-factor correlations remained below the advised limit of 0.85, affirming strong discriminant validity. The corresponding CR, AVE, and inter-factor correlation coefficient values are reported in Table 8.

**Table 8:** SEQ-C discriminant validity.

Variable	Anger	Anxiety	Dejection	Excitement	Happiness
Anger	**0.796**				
Anxiety	0.533**	**0.761**			
Dejection	0.534**	0.519**	**0.799**		
Excitement	0.574**	0.560**	0.496**	**0.758**	
Happiness	0.568**	0.559**	0.561**	0.524**	**0.738**

The diagonal bold is the AVE square root value, and the lower triangle is the Pearson correlation of factors. **Correlation is significant at the 0.01 level (two-tailed).

## Discussion

The present study aimed to examine the psychometric properties of the Chinese version of the Sport Emotion Questionnaire (SEQ-C) among adolescent table tennis athletes. The results demonstrated that the SEQ-C exhibited a stable five-factor structure consistent with the original SEQ, including anger, anxiety, dejection, excitement, and happiness. Exploratory factor analysis (EFA) supported the extraction of five factors, explaining a substantial proportion of the total variance. Furthermore, confirmatory factor analysis (CFA) indicated a satisfactory model fit (RMSEA = 0.042, SRMR = 0.026, CFI = 0.971, TLI = 0.966). All items showed strong factor loadings, and the construct reliability (CR) and average variance extracted (AVE) values met the recommended thresholds. These findings suggest that the SEQ-C demonstrates good reliability and validity and can serve as an effective instrument for assessing emotional states among Chinese adolescent table tennis athletes.

The results of the present study are generally consistent with previous validation studies of the SEQ conducted in different cultural contexts. For example, Arnold and Fletcher (2015) confirmed the five-factor structure of the SEQ among athletes in the United Kingdom, while Dehkordi et al. (2020) and Gomes et al. (2021) reported similar findings in Persian and Portuguese samples. These studies consistently demonstrated that the five emotional dimensions of anxiety, anger, dejection, excitement, and happiness adequately capture athletes’ emotional experiences before competition. The findings of the current study further support the cross-cultural stability of the SEQ structure, indicating that the five-dimensional emotional framework can also be effectively applied to Chinese adolescent athletes.

However, some previous studies have reported variations in the latent structure of the SEQ. For instance, Bayköse and Şakar (2018) proposed a four-factor structure when validating the scale among Turkish athletes. Similarly, Hagan et al. (2022) suggested a hierarchical structure when examining athletes from several African countries. Compared with these studies, the present research retained the original five-factor structure without removing any items. This difference may reflect variations in sample characteristics, research design, and cultural context.

Several factors may explain the differences observed between the present study and previous research. First, cultural background may influence how athletes experience and express emotions. Chinese cultural traditions emphasize emotional moderation and self-regulation, which may shape the emotional patterns of athletes. Second, the sample characteristics in this study were relatively homogeneous, consisting of adolescent table tennis players aged 15–18 years who trained in a highly structured sports environment. Such training contexts may produce more stable emotional patterns compared with more heterogeneous athlete populations. Finally, methodological differences, such as sample size, statistical techniques, and measurement procedures, may also contribute to variations in the factor structure across studies.

The findings of this study have both theoretical and practical implications. From a theoretical perspective, the validation of the SEQ-C provides further evidence supporting the cross-cultural applicability of the SEQ framework in sport psychology research. It also contributes to the understanding of emotional assessment among adolescent athletes within the Chinese cultural context. From a practical perspective, the SEQ-C offers coaches, sport psychologists, and researchers a reliable tool for assessing athletes’ emotional states during training and competition. By identifying emotional patterns such as anxiety, anger, or excitement, practitioners may develop more targeted psychological interventions and emotional regulation strategies to support athletes’ performance and well-being.

### Research limitation

There were some limitations in this study. Firstly, the participants were limited to young table tennis athletes aged 15–18 from one district in China, which restricts the generalizability of the findings to the broader population of Chinese athletes. Therefore, future studies should include a more diverse and representative sample, incorporating athletes from different sports disciplines, various age groups (e.g., youth or adult athletes), and different competitive levels (e.g., amateur, professional, or elite athletes) to enhance the applicability of the results. In addition, although the participants were all table tennis players, they represented different competitive levels and training experiences. Such heterogeneity within the sample may influence athletes’ emotional responses and potentially affect the representativeness of the findings. Future studies should consider recruiting more homogeneous samples or conducting subgroup analyses based on competitive level to better control for potential differences in athletic identity and performance goals. Secondly, all data were collected through self-report questionnaires, without additional forms of corroboration. Self-reported data can have a high degree of autonomy and may be influenced by social desirability bias, where participants tend to respond in ways that portray themselves more favourably (He and van de Vijver, 2013). To minimize this bias, participants were instructed to respond as honestly as possible. Thirdly, the study employed a cross-sectional research design, which restricts the ability to observe changes in athletes’ emotional states over time. Future research should consider longitudinal approaches to more accurately capture the development and evolution of athletes’ emotional experiences, thereby offering coaches more targeted and practical guidance. Additionally, future studies should conduct measurement invariance tests to examine whether the SEQ-C demonstrates structural consistency across different genders and age groups in China. This will provide further evidence on the validity of the scale and cross-group applicability.

## Conclusion

In conclusion, this study showed that the items from the original English version of the SEQ could support the validity and reliability of the SEQ-C among Chinese adolescent table tennis athletes. Therefore, with the SEQ-C, researchers, sport psychologists, and coaches would have an instrument with accurate translation and reliability to do so. Moreover, the SEQ-C can capture the unique emotional characteristics experienced by athletes in more realistic pre-competition settings, offering valuable insights for emotional regulation and intervention in sports contexts.

## Data Availability

The datasets presented in this study can be found in online repositories. The names of the repository/repositories and accession number(s) can be found in the article/supplementary material.
